# Supplementary material for: All-optical control of phase singularities using strong light-matter coupling
Source: Nat Commun. 2022 Apr 5;13:1809. doi: 10.1038/s41467-022-29399-x (PMC8983677; doi:10.1038/s41467-022-29399-x)
Supplement: Supplementary file 1 — Supplementary Information [file 41467_2022_29399_MOESM1_ESM.pdf]

Supplementary Information for:

All-optical control of phase singularities using strong light-matter coupling

Philip A. Thomas, Kishan S. Menghrajani and William L. Barnes

Department of Physics and Astronomy, University of Exeter,
Exeter, EX4 4QL, United Kingdom

Contents

S1. SPI/MC chemical structure and MC transmittance spectrum **2**

S2. Ψ spectra for a range of film thicknesses **3**

S3. Emergence of phase singularities for strongly coupled TE modes **4**

S1. SPI/MC chemical structure and MC transmittance spectrum

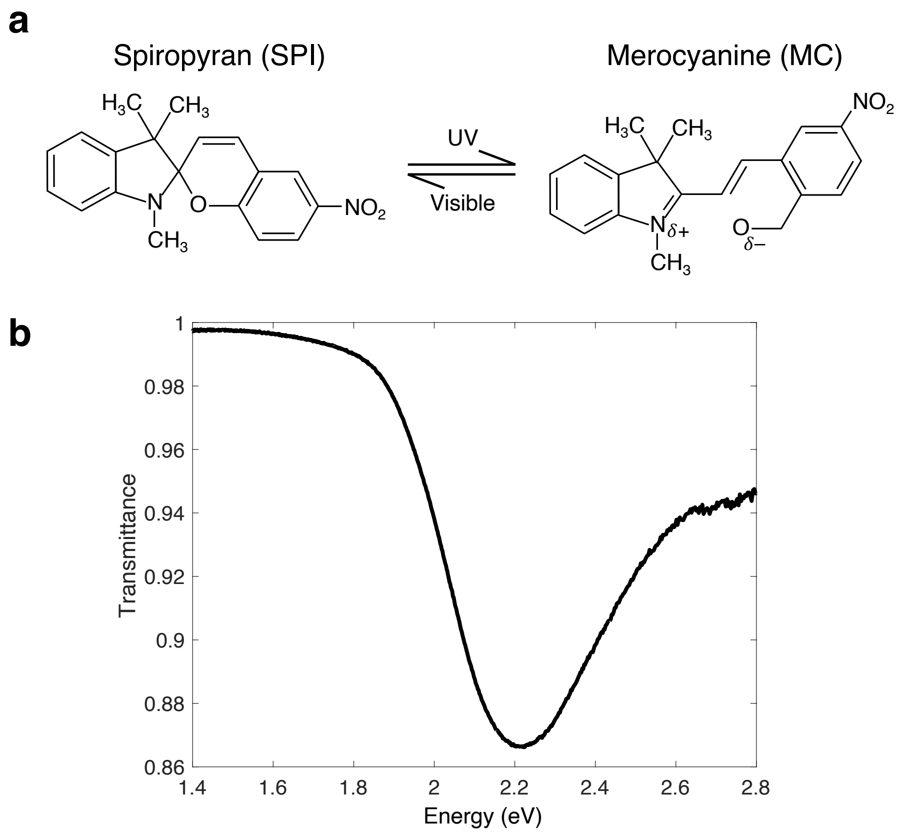

Figure S1: (a) Spiropyran and merocyanine chemical structures. (b) Transmittance through a merocyanine (MC) film (thickness 150 nm) spin-coated on a glass substrate, normalised against transmission for an uncoated substrate (see Methods for fabrication).

S2. Ψ spectra for a range of film thicknesses

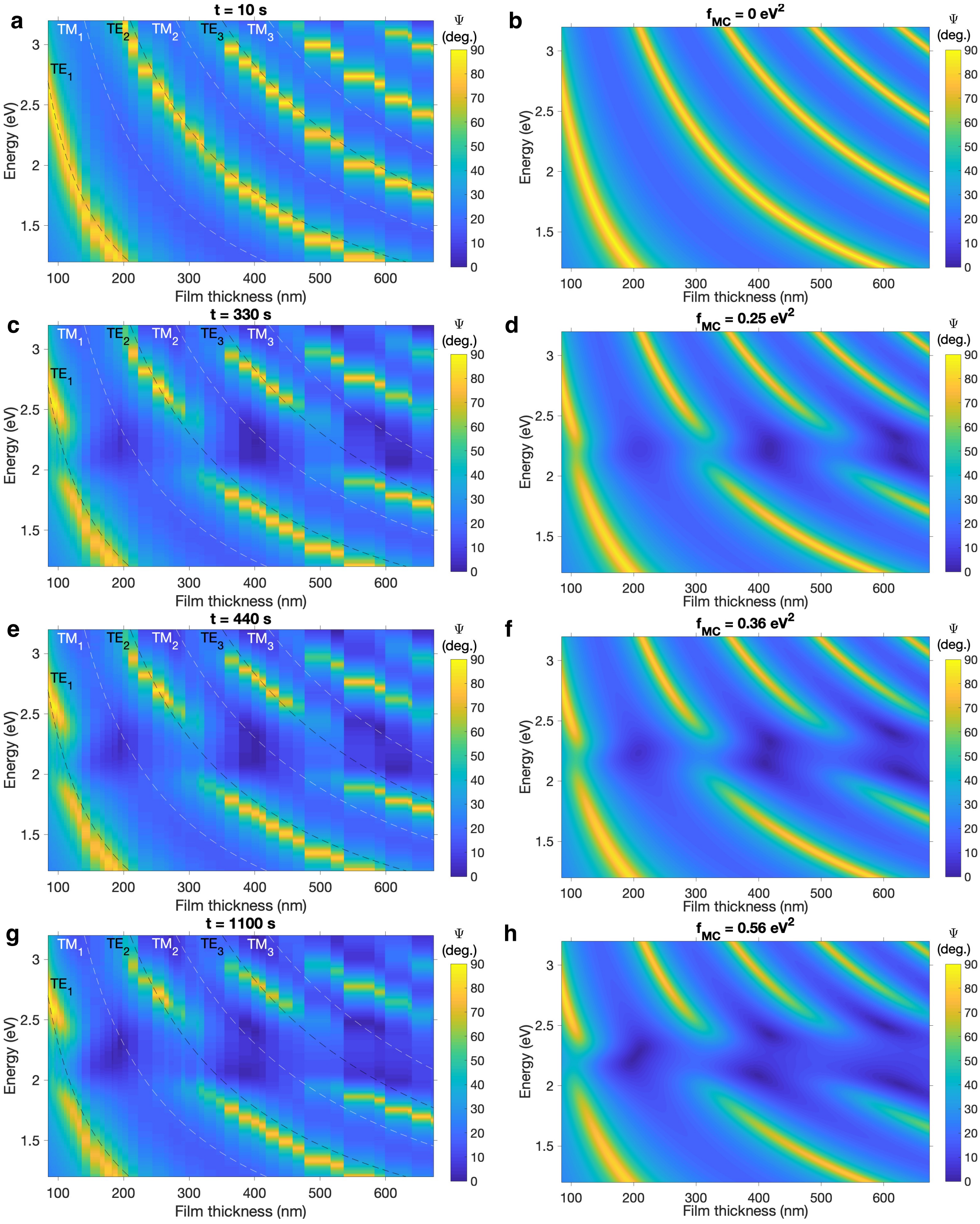

Figure S2: (a,c,e,g) Experimental and (b,d,f,h) calculated (Fresnel approach) dispersion plots constructed using the ellipsometric parameter Ψ for SPI/MC films over a range of thicknesses at fixed angle $\theta = 65°$. The MC concentrations were varied in experimental plots by UV exposure time ((a) $t = 10$ s, (c) $t = 330$ s, (e) $t = 440$ s, (g) $t = 1,100$ s) and in calculated plots by varying the Lorentz oscillator strength of MC ((b) $f_{\mathrm{MC}} = 0$ eV^2, (d) $f = 0.25$ eV^2, (f) $f = 0.36$ eV^2 and (h) $f = 0.56$ eV^2). In panels (a,c,e,g) the positions of the uncoupled TE modes are indicated by the black dashed lines and the positions of the uncoupled TM modes are indicated by the white dashed lines. This Figure is the

amplitude counterpart to Main Figure 3.

S3. Emergence of phase singularities for strongly coupled TE modes

In the main manuscript we observed the creation of phase singularities when TM leaky modes strongly coupled to the MC molecular resonance. We did not observe the creation of new phase singularities arising from the strong coupling of TE leaky modes and the MC molecular resonance.

Here we seek to answer two questions:

1. Why did we observe the creation of phase singularities associated with strongly coupled TM modes but not with TE modes? (Even though the coupling between TE modes and the MC resonance is stronger than between TM modes and the MC resonance.)

2. Is it possible to create phase singularities by coupling TE leaky modes to the MC resonance?

To answer the first question, in Figure S2 we plot ellipsometric parameters (calculated using the transfer matrix method) for SPI films over a wide energy range while varying the angle of incidence θ. At $\theta = 15°$ (Fig. S2a-b) we observe phase singularities for each TE leaky mode. At such a shallow incident angle the phase singularities in each pair occur at very similar energies; their energies closely match the energy ($E = 4.45$ eV) at which our Cauchy model for SPI predicts that the SPI film and Si substrate permittivities match ($\epsilon_{\mathrm{SPI}} = \epsilon_{\mathrm{Si}}$). Increasing the incident angle to $\theta = 40°$ (Fig. S2c-d) increases the separation between each pair of phase singularities: the higher energy phase singularities are shifted to even higher energies and the lower energy phase singularities are shifted to even lower energies. Increasing θ from 40° to 60° (Fig. S2e-f) shifts the higher energy phase singularities outside our plotted range while the lower energy phase singularities have been shifted to 2.8 eV. Increasing θ to 65° shifts the lower phase singularity outside our plotted range. These results are consistent with our experimental results in Main Figure 3a (for which $\theta = 65°$) to within a few degrees. The slight mismatch between experiment and calculation in predicting the phase response is likely due to our choice of model of the permittivity of SPI. Our simple Cauchy model (see Methods) accurately describes the behaviour of SPI well in the spectral region 1.2 eV $< E <$ 3.2 eV (apart from the precise conditions for the phase singularity), but is inadequate in describing its UV response and hence predicting where $\epsilon_{\mathrm{SPI}} = \epsilon_{\mathrm{Si}}$.

To summarise our answer to the first question, we did not observe the creation of new phase singularities arising from the strong coupling of TE leaky modes and the MC molecular resonance because at $\theta = 65°$ pairs of TE phase singularities already exist in SPI films but cannot be fully observed in the plotted experimental range in Main Figure 3.

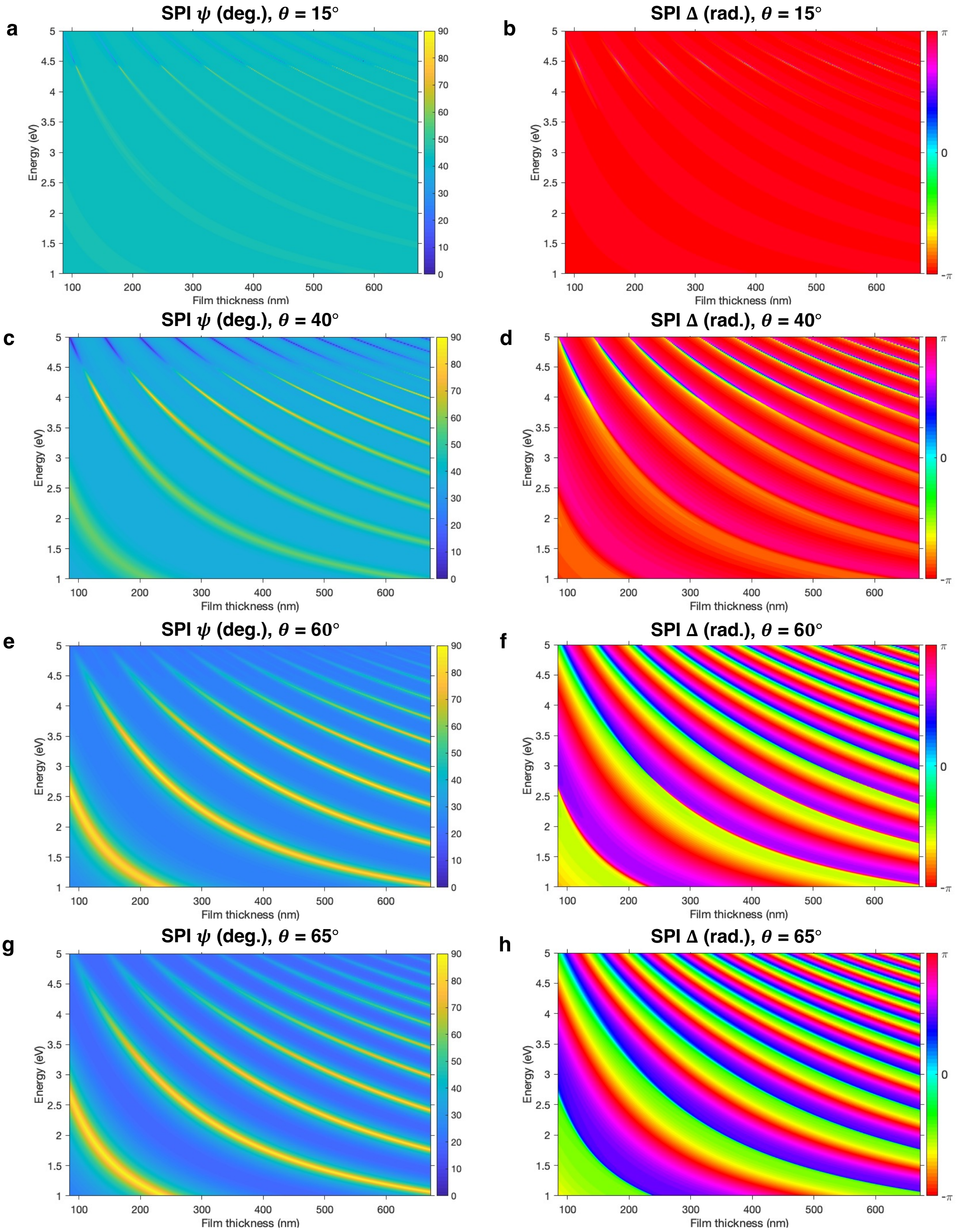

Figure S3: Calculated ellipsometric parameters Ψ and Δ for SPI films over a range of thicknesses for incident angles of (a,b) 15°, (c,d) 40°, (e,f) 60° and (g,h) 65°.

To answer the second question, we first note that we conducted our experiments at a high incident angle of $\theta = 65°$ to generate the confined electromagnetic fields necessary for the creation of TM phase singularities. At such a high incident angle, pairs of TE phase singularities have already been created but cannot be fully observed in the plotted experimental range in Main Figure 3. TE leaky modes generate stronger confined electromagnetic fields than TM leaky modes and electromagnetic field confinement decreases with lower incident angle[1, 2]. We therefore expect to see the creation of phase singularities associated with strong coupling between TE modes and the MC resonance at a lower incident angle. This is confirmed in Figure S3, where we have calculated the ellipsometric parameters for MC films at $\theta = 40°$ and varied the MC resonance Lorentz oscillator strength. There are many similarities to the phase response of TM modes as they undergo strong coupling (Manuscript Figure 3). As the Lorentz oscillator strength of the MC resonance is increased, pairs of phase singularities appear as each TE mode couples to the MC resonance. Higher order TE leaky modes (which match the MC resonance for thicker films) are better confined than lower order leaky modes. Therefore, as we gradually increase oscillator strength we first observe the anticrossing indicative of strong coupling in Ψ (and the corresponding phase singularity creation in Δ) for higher-order TE modes. However, unlike the TM case in Main Figure 3, here we observe the creation of two pairs of phase singularities as each TE mode strongly couples with the MC resonance. We first observe the creation of a phase singularity pair as each TE leaky mode is first perturbed by the MC resonance in the weak coupling regime. Then, as anticrossing becomes clear in Ψ, a second pair of phase singularities appears. These second pairs of phase singularities, approximately corresponding to the ends of each polariton branch at the strong coupling-induced photonic stop-band, behave in the same manner as the phase singularities observed in Main Figure 3.

To summarise our answer to the second question, it is possible to create phase singularities by coupling TE leaky modes to the MC resonance when the angle of incidence is decreased. When TE modes strongly couple to the MC resonance it is possible to observe the creation of two pairs of phase singularities per leaky mode (instead of just one pair per leaky mode, as is the case for TM modes).

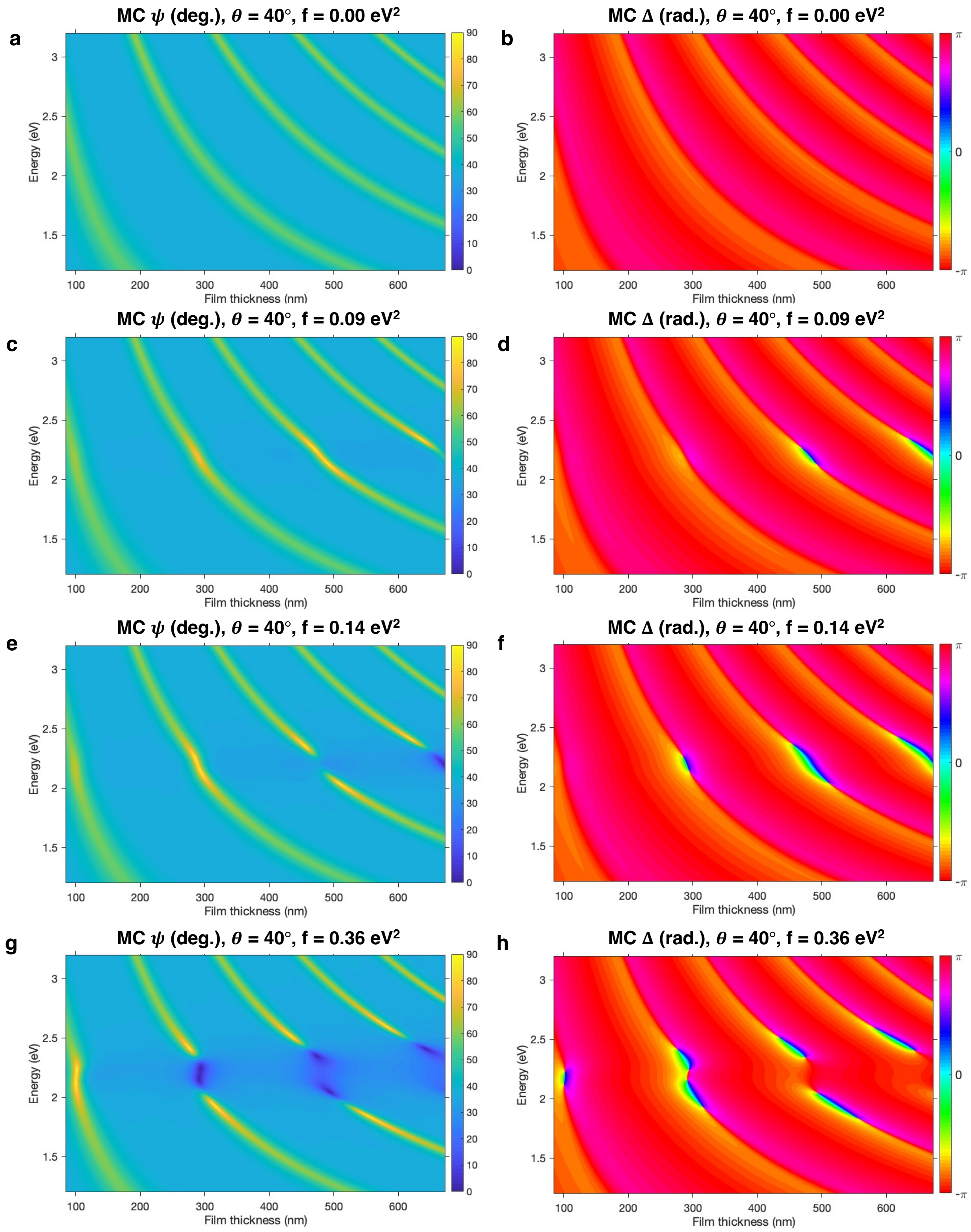

Figure S4: Calculated ellipsometric parameters Ψ and Δ for MC films over a range of thicknesses at an incident angles of 40° with the MC Lorentz oscillator strength set to (a,b) 0 eV^2, (c,d) 0.09 eV^2, (e,f) 0.14 eV^2 and (g,h) 0.36 eV^2.

References

[1] Hecht, E. *Optics* (5th edition, Addison Wesley, 2002.)

[2] Thomas, P. A., Menghrajani, K. S. & Barnes, W. L. Cavity-Free Ultrastrong Light-Matter Coupling. *J. Phys. Chem. Lett.* **12**, 6914–6918 (2021). DOI: 10.1021/acs.jpclett.1c01695
